# The dual role of microRNA (miR)-20b in cancers: Friend or foe?

**DOI:** 10.1186/s12964-022-01019-7

**Published:** 2023-01-30

**Authors:** Ahmet İlhan, Shayan Golestani, Seyyed Ghavam Shafagh, Fatemeh Asadi, Danyal Daneshdoust, Bashar Zuhair Talib Al-Naqeeb, Mohammed Mahdi Nemati, Fateme Khalatbari, Amirhossein Fakhre Yaseri

**Affiliations:** 1 Department of Medical Biochemistry, Faculty of Medicine, Cukurova University, Adana, Turkey; 2 Department of Oral and Maxillofacial Surgery, Dental School, Islamic Azad University, Isfahan (Khorasgan) Branch, Isfahan, Iran; 3 Faculty of Medicine, Iran University of Medical Sciences, Tehran, Iran; 4 Department of Genetics, Marvdasht Branch, Islamic Azad University, Marvdasht, Iran; 5 School of Medicine, Babol University of Medical Sciences, Babol, Iran; 6 Anesthesia Technology Department, Al-Turath University College, Al Mansour, Baghdad, Iraq; 7 Faculty of Medicine, Urmia University of Medical Sciences, Urmia, Iran; 8 Department of Pathology, Mashhad Medical Sciences Branch, Islamic Azad University, Mashhad, Iran; 9 Department of Genetic, Faculty of Medicine, Qazvin University of Medical Sciences, Qazvin, Iran

**Keywords:** miRNAs, Cancer, miR-20b, Apoptosis

## Abstract

MicroRNAs, as non-coding transcripts, modulate gene expression through RNA silencing under normal physiological conditions. Their aberrant expression has strongly associated with tumorigenesis and cancer development. MiR-20b is one of the crucial miRNAs that regulate essential biological processes such as cell proliferation, apoptosis, autophagy, and migration. Deregulated levels of miR-20b contribute to the early- and advanced stages of cancer. On the other hand, investigations emphasize the tumor suppressor ability of miR-20b. High-throughput strategies are developed to identify miR-20b potential targets, providing the proper insight into its molecular mechanism of action. Moreover, accumulated results suggest that miR-20b exerts its effects through diverse signaling pathways, including PI3K/AKT/mTOR and ERK axes. Restoration of the altered expression levels of miR-20b induces cell apoptosis and reduces invasion and migration. Further, miR-20b can be used as a biomarker in cancer. The current comprehensive review could lead to a better understanding of the miR-20b in either tumorigenesis or tumor regression that may open new avenues for cancer treatment.

**Video Abstract**

**Supplementary Information:**

The online version contains supplementary material available at 10.1186/s12964-022-01019-7.

## Introduction

Investigations in the recent two decades changed the researchers' point of view from mRNA to non-coding RNAs as a pivotal regulator of the human genome [1]. The term "non-coding" in biology generally refers to RNA that does not translate to protein. In mammalian genomes, approximately 97% of the genome is transcribed as non-coding RNA, including transfer RNA (tRNA), ribosomal RNA (rRNA), circular RNA, transposons, and microRNAs [2, 3].

MicroRNAs (miRNAs) represent short non-coding RNAs binding to the 3′ untranslated region (UTR) of specific mRNA and suppressing its expression [4]. On the other hand, recent investigations have implied that miRNAs can interact with 5′ UTR of mRNA, promoter of genes, and protein to exert their effects [5]. Indeed, they profoundly impact the transcription and translation of genes through either inhibition or induction. MiRNAs subsequently modulate a wide range of cellular functions [6]. Interaction between miRNAs and other non-coding RNAs like circular RNA to control cellular function make miRNAs essential cell regulators [7]. Furthermore, the comprehensive analysis estimated that one-third of the human genome is modulated through miRNAs [8]. It seems reasonable that impaired miRNA expression may underly human diseases such as cancer.

Cancer is a complex disease comprising uncontrolled growth and division of cells to form tumors in part of the body [9]. Tumor cells can spread to other organs and create a secondary tumor called metastasis [10]. Multiple cell effectors, such as miRNAs, are associated with tumorigenesis [11]. A broad spectrum of reports has evinced that the aberrant expression of miRNAs may cause cancer. Indeed, miRNA levels vary in tumor cells because of diverse mechanisms, including disruption in miRNA biogenesis, deletion and amplification of related genes, dysregulation in transcriptional control mechanisms, and abnormal epigenetic changes [12]. In the next step, miRNA-mediated gene modulation and underlying signaling pathways are disrupted [13]. Then these effects are traceable at the cellular levels, where cell functions may be impaired, and symptoms of the diseases appear. The miRNAs are classified into tumor suppressors and oncogenes according to their regulatory role in target genes [14]. Loss of tumor suppressor miRNAs facilities tumor formation and progression by up-regulating oncogenes expression and activation. In contrast, enhanced levels of oncogenic miRNAs down-regulate tumor suppressor genes and result in malignancy.

IsomiRs refer to non-canonical variants of the reference miRNA that can differ in sequence, length, or both [15]. The miRNAs like miR-20a and miR-20b with one or two nucleotide differences are denoted with a lowercase letter. Besides, based on the origin of sequence from opposite arms of pre-miRNA, mature miRNA is annotated with a -5p or -3p, such as miR-20b-5p [16]. The most important of these findings is that the functions and biological activity of isomiRs are diverse from each other and canonical miRNA [17].

MiR-20a is a member of the miR-17-92 cluster and is located on chromosome 13q31.3, while miR-20b belongs to the miR-106b-25 cluster and is found on chromosome Xq26.2 [18]. Based on different gene regulatory elements, the expression pattern of these two clusters varies in a cell- and tissue-dependent manner. On the other hand, both of these clusters are called the miR-17 family [19]. Members of this family appear to be the result of gene duplication occurrences. The seed sequence (AAAGUG) of members, despite minor differences in length and nucleotide composition, is the same which suggests functional redundancy [20]. As a result, miR-20a and miR-20b have different targets and regulate a variety of downstream biological processes. Plenty of studies have reviewed the miR-20a role in a variety of malignancies, including thyroid cancer, gastric cancer, gliomas, cervical cancer, colon adenocarcinoma, and prostate cancer [21].

The miR-20b is a well-studied regulator in healthy and pathological human conditions (Table 1). Cumulative evidence has illustrated that the miR-20b is involved in normal development, aging, immune diseases, neurodegenerative diseases, and cardiovascular diseases via cell cycle regulation accompanied by moderating cell proliferation and apoptosis [22–24]. Besides, animal modeling has been performed to study miR-20b function in healthy human physiology and related disorders (Table 2). Due to the extensive scope of this topic, we focused on miR-20b potential role in tumorigenesis and cancer development.

**Table 1:** The miR-20b role in modulating the biological function of human cells

Cell type	Biological process	Signal pathway	Target genes	Potential diseases	References
Vascular smooth muscle cells	Blood pressure	–	MAGI3	Hypertension	[25]
Endothelial cells	Autophagy	HIF-1	ULK1	–	[26]
Placental trophoblast cells	Viability	–	MCL-1	Preeclampsia	[27]
Smooth muscle cells	Proliferation	STAT3	PDGF	Asthma	[28]
Trophoblast cell	Migration	–	MMP-2	Preeclampsia	[29]
T Cell	Proliferation and Activation	NFAT	NFAT5 and CAMTA1	Myasthenia Gravis	[30]
Mesenchymal stem cells	Differentiation	Notch	Ngn2, MAP2, and TUBB3	–	[31]
Lung tissues cells	Apoptosis	Mitochondrial intrinsic pathway	MFN1 and MFN2	Lung injury	[32]
Cardiac cell	Apoptosis and Differentiation	BMP	–	Heart diseases	[33]
Chondrocyte	Proliferation and autophagy	PI3K/AKT/mTOR	ATG10	Osteoarthritis	[34]
Neurons	Differentiation	WNT	NRSF/REST	Neurological disorders	[35]
Macrophage-derived foam cells	Cholesterol metabolism	–	ABCA1	Atherosclerosis	[36]
pluripotent stem cells	Apoptosis	Intrinsic pathway	BIM	–	[37]
Hematopoietic stem cell	Differentiation	–	MAFB	–	[38]
Macrophage and kidney	Lipid metabolism	Epigenetic pathways	ABCA1, ABCG1, and SCARBI	Atherosclerosis	[39]
Multipotent Stromal Cells	Cell cycle and DNA synthesis	–	p21, CCND1, and E2F1	–	[40]
Human umbilical vein endothelial cells	Cell senescence	Wnt/beta-catenin	TXNIP	Cardiovascular disease	[41]
Cardiomyocytes	Apoptosis	NF-kappa B	HIF-1 alpha	Cardiovascular disease	[23]

**Table 2:** The miR-20b as a regulator in animal models of diseases

Species	Model of disease	Biological process	Target genes	References
Fish	Mitochondrial	Metabolism	Cytochrome c oxidase	[42]
	Immunity	Inflammation	TRAF6	[43]
Mice	Lung	Embryonic development	CircRNAs	[44]
	Osteoarthritis	Apoptosis	HOTAIR	[45]
	Infection	Inflammation	NLRP3	[46]
	Chronic asthma	Inflammation	TGF-beta	[47]
Rat	kidney	Inflammation	ATG7 and TLR4	[48]
	Chronic injury	Immune function	Akt3	[49]
	Acute pancreatitis	Inflammation, apoptosis, and angiogenesis	Akt3	[50]
	Ischemia–reperfusion injury	Metabolism	Smad	[51]
	Intestinal dysfunction	Inflammation	DMNT3B	[52]

In contrast to its tumor-supportive role, miR-20b could elicit anti-tumor responses, thus decreasing tumor progression. Based on miR-20b levels and its various targets in a wide range of signaling pathways, major cellular processes such as proliferation, cell cycle, apoptosis, autophagy, and migration are affected. As a result, its role can be different. Aberrant expression of miRNAs has been noted as a molecular tool for the diagnosis and prognosis of cancer. Indeed, surveillance of tumor markers may have advantages for patients before the onset of symptoms, during, and after treatment. Several studies have revealed that miR20b is found in biological fluid and tissue specimens, which have potential applications as biomarkers. These findings are essential to clarifying the therapeutic and diagnostic role of miR-20b in cancer.

## Approaches to determine the targets of miR-20b

Identifying potential candidate genes targeted through miRNAs is essential to discovering the function of microRNAs in gene regulatory networks. As a consequence, miRNA-related cellular processes can be determined. It can be considered a pivotal procedure to illuminate the pathogenesis of cancer. In the following, different approaches to predicting targets of miR-20b are introduced.

### The miR-20b in normal physiological condition

Growing evidence has revealed the fundamental roles of miRNAs in normal physiological conditions [53]. In this regard, miRNAs act toward maintaining cell function and homeostasis through cooperation with other cellular components and plenty of effectors. It has been authenticated that miR-20b is involved in the regulatory genes network within human cells. In Table 1, we summarized the findings from related investigations around the regulatory role of miR-20b in human biological specimens and cell lines. Evidence has indicated that fundamental cellular processes such as apoptosis, proliferation, differentiation, and T-cell activation are regulated upon miR-20. On the other hand, certain levels of miR-20b in cells are associated with their required function. As a result, changes in miR-20b levels lead to dysregulation of vital biological functions, which triggers human diseases.

### Evolutionarily conserved miR-20b gene and comparative biology

Comparative biology is an approach that examines the differences and similarities between organisms, especially in biology, biochemistry, genetics, and physiology [54]. Conserved intra- and inter-species genes and proteins are of interest in comparative biology as a conserved gene in one organism may have a similar function in another [55]. MiRNAs are mentioned as the conserved genes during evolution [56]. Therefore, studies on animal models provide valuable information about the gene-miRNA network, leading to a further understanding of specific miRNA functions in human physiology. According to Table 2, miR-20b can modulate biological function in different animal models of diseases. Considering affected potential targets of miR-20b in the animal models might give a clue to its mechanism of action in humans.

### High-throughput analysis to predict miR-20b potential target

Nowadays, novel approaches are established to determine the miRNA-mRNA interaction and underlying mechanisms of action. A computational method is a mathematical approach to merging biology, chemistry, and computer science [57]. This high-throughput analysis can accurately describe the miRNA-related target genes in various diseases. Using computational methods, Kim et al. determined that miR-20b targets transcription factor E2F1 in breast cancer [58]. E2F1 is a transcription factor that facilitates the synthesis of DNA and cell cycle in mammalian cells [59]. Consequently, targeting E2F1 by miR-20b inhibits tumor cell division. Integrative computational algorithms can analyze dysregulated miRNA targets in autoimmune diseases to identify strong candidate genes. Significantly, miR-20b has been determined as a modulator of helper T-cells differentiation with a high score [60]. Negative and positive regulators of T-cells differentiation, such as FOXO1 (Forkhead Box O1) and STAT3 (Signal transducer and activator of transcription 3), have been defined as significant miR-20b downstream target genes.

### The miR-20b and cancer

**Fig. 1:**
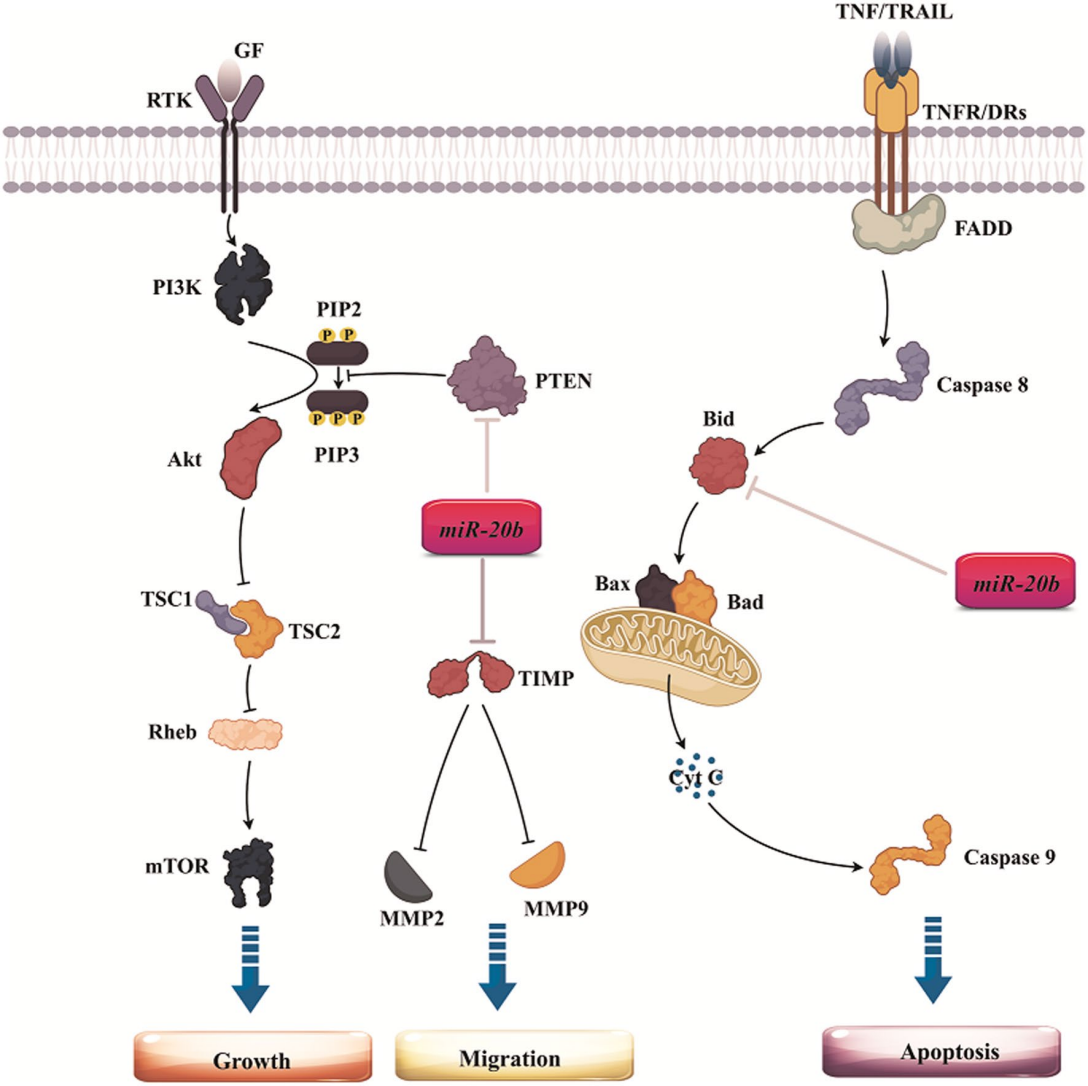
Tumor-supportive role of miR-20b by stimulating tumor cell growth and migration while inhibiting their apoptosis

**Fig. 2:**
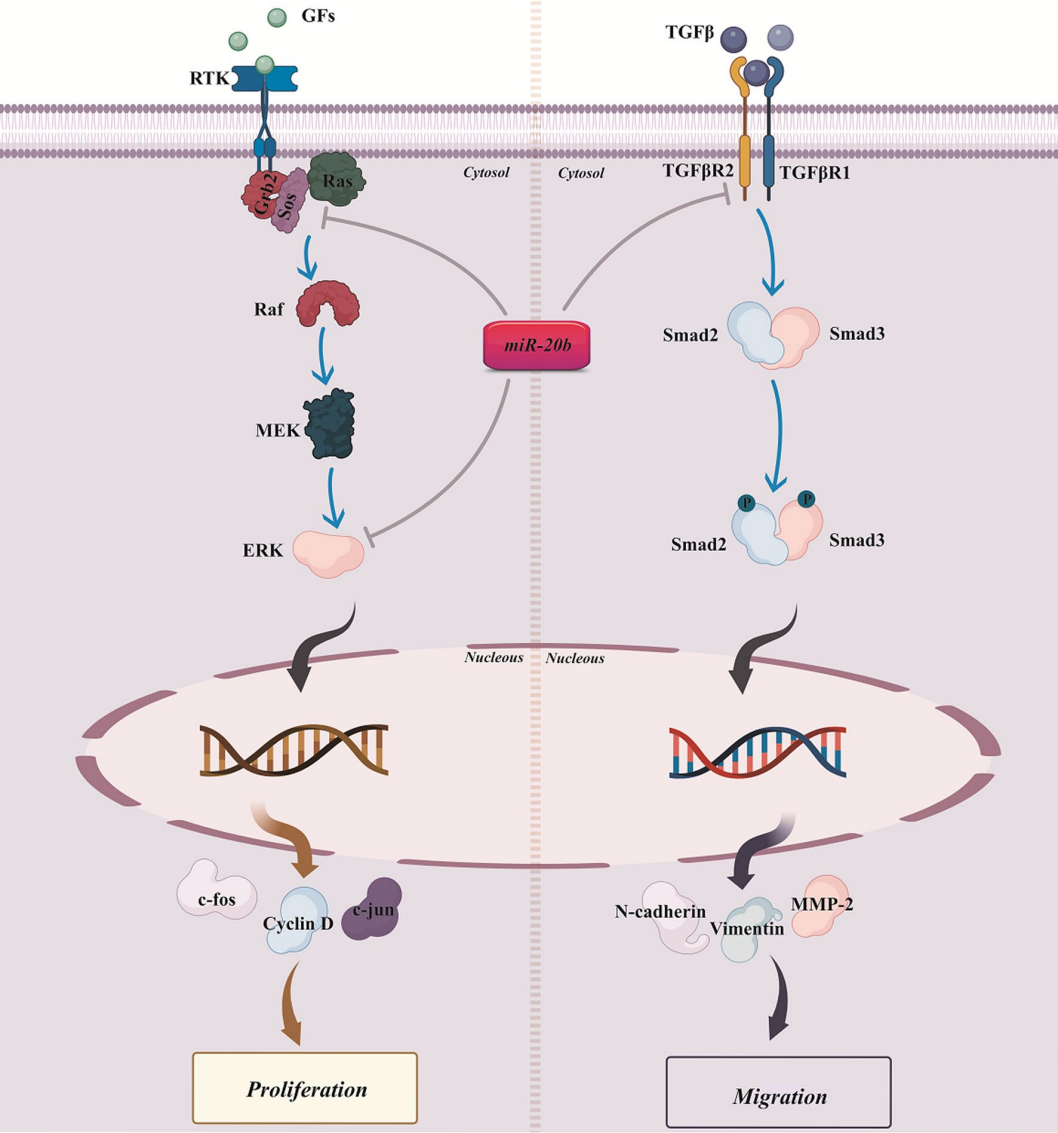
Tumor-suppressor role of miR-20b by inhibiting tumor cell growth and migration

Aberrant expression patterns of miRNAs influence the critical properties of tumors, such as escaping growth inhibitors, uncontrolled growth, promoting migration and invasion, preserving proliferative signaling pathways, suppressing apoptosis, and activating angiogenesis [12]. In contrast to the tumor-promoting role of miRNAs, these non-coding RNAs can act as tumor suppressors and inhibit tumor formation and progression under certain conditions. The miRNAs exert their effects through target genes and related signaling pathways. As mentioned, miR-20b binds to various targets and regulates signaling pathways and biological processes. Over the past years, much more information has become available on the occurrence of cancer upon disruption in miR-20b function and levels (Table 3). We reviewed the role of miR-20b in different solid tumors with emphasis on either its tumor-supportive (Fig. 1) or suppressor (Fig. 2) activities.

**Table 3:** The biological role of miR-20b in various cancer and its targets

Type of cancer	Levels in tumor	Biological process	Target gene	Description of target gene	References
Breast	Increased	Proliferation and cell cycle	PTEN and BRCA1	Tumor suppressor	[61]
	Decreased	Metastasis	VEGF	Angiogenesis	[62]
	Increased	Autophagy	RB1CC1/FIP200	Autophagosome formation-related proteins	[63]
	Increased	Proliferation and cell cycle	CCND1 and E2F1inhibitors	Tumor suppressor	[64]
Ovarian	Increased	Apoptosis	Bcl2	Anti-apoptotic	[65]
	Decreased	cell cycle	Cyclin D	Oncogene	[66]
Prostate	Increased	proliferation and cell cycle	PTEN	Tumor suppressor	[67]
	Decreased	Metastasis	TGFBR2	Oncogene	[68]
Cervical	Increased	Metastasis	TIMP-2	MMPs inhibitor	[69]
Lung	Increased	Proliferation and cell cycle	BTG3	An anti-proliferative protein	[70]
Esophageal	Increased	Metastasis	PTEN	Tumor suppressor	[71]
Colon	Increased	Drug resistance	ADAM9	MMPs activator	[72]
	Increased	Metastasis	Oct4 and MALAT1	Cancer stem cell regulators	[73]
	Increased	Migration and invasion	PTEN	Tumor suppressor	[74]
	Decreased	Drug resistance	Syndecan-2	Promote cell survival	[75]
Bladder	Decreased	Metastasis	MMP2	Oncogene	[76]
	Decreased	Proliferation	CDK6, CDK2, and cyclin D	Cell cycle-regulated genes	[76]
Thyroid	Decreased	Proliferation	Sos1 and ERK2	Oncogene	[77]
	Decreased	Apoptosis	DUXAP8	As long non-coding RNA	[78]
Liver	Increased	Proliferation	PTEN	Tumor suppressor	[79]
	Decreased	Drug resistance	CDC37L1	A cochaperone protein leading to cell survival	[80]
	Increased	Angiogenesis	STAT3	Suppress VEGF expression	[81]
kidney	Decreased	Apoptosis	MAPK	Inhibit apoptosis	[82]
Gastric	Decreased	Drug resistance	HIF1A	Up-regulates drug resistance-related genes	[83]
Endometrial	Decreased	EMT	HIF-1 alpha	Induce hypoxia	[84]

### Proliferation and cell cycle

Cell division is strongly associated with tumorigenesis and counted as an essential hallmark of malignancy. At the molecular levels, extra- and intracellular signaling pathways, growth factors, and bona fide of hormones keep an equilibrium between the induction of cell division and its inhibition [12]. Abnormal activity of these regulatory pathways leads to cancerous cells with out-of-control proliferation. Various studies have assessed the efficacy of miR-20b in cell division and cell cycle. It significantly integrates into several vital cell proliferation pathways, and the improper modulation of this miRNA is responsible for preserving proliferative signaling and escaping growth inhibitors in tumor cells.

The miR-20b is overexpressed in MCF-7 and MDA-MB-231 cell lines compared to healthy cells [85]. It may refer to the potential role of miR-20b in breast cancer tumorigenesis. Olga et al. have shown that radiation increases the levels of miR-20b, which targets the Phosphatase and tensin homolog (PTEN) and Breast cancer type 1 (BRCA1) genes, and promotes the proliferation and cell cycle of breast cancer cells [86]. BRCA1 is involved in homologous recombination, a kind of DNA repair mechanism. Impairment in BRCA1 normal activity increases genomic instability and facilitates cancer cell proliferation and tumor progression. Besides, incompetent DNA repair in cancer cells yields increased proliferation. Indeed, miR-20b triggers genomic instability and elevated cell proliferation by targeting BRCA1. The growing evidence suggests that miR-20b broadly targets PTEN in multiple cancer cells. Up-regulated miR-20b negatively correlates with PTEN expression in breast cancer specimens [87]. PTEN serves as a tumor suppressor, tightly deterring transformed cell growth and division [88]. Besides, aberrant expression of miR-20b is closely related to prostate cancer [89]. MiR-20b is elevated in prostate cancer cells and triggers their proliferation. Bioinformatics analysis showed that the PTEN gene is a potential target of miR-20b, as verified by preclinical experiments [67]. Furthermore, it has been indicated that miR-20b leads to hepatocellular and colorectal cancer cell proliferation through binding PTEN and down-regulates its expression [74, 79]. Using the antagomir of miR-20b enhances PTEN levels and causes tumor regression. Antagomirs are synthetic antisense oligonucleotides that bind to the desired miRNA and reduce or block its activity [90].

Overexpression of miR-20b positively correlates with poor prognosis in lung cancer [91]. To understand the working mechanism of miR-20b in lung cancer proliferation, Xuan et al. have demonstrated that miR-20b is up-regulated in non-small cell lung cancer cells [70]. Further, molecular experiments revealed that B-cell translocation gene 3 (BTG3) reduced upon miR-20b overexpression and led to tumor cell proliferation [70]. BTG3 is an anti-proliferative protein that loses its normal function during tumorigenesis in human cancers [45]. The level of miR-20b and its function integrate into proliferation-related signaling pathways. Ectopic low-level of miR-20b is associated with tumorigenesis and progression of thyroid carcinoma [77]. It directly binds to extracellular signal-regulated kinase 2 (ERK2) and son of sevenless homolog 1 (SOS1) [77]. These proteins are member of the mitogen-activated protein kinase (MAPK) signaling pathway, which promote cellular proliferation and differentiation [92].

Despite the oncogenesis effect of miR-20b in promoting cell proliferation, miR-20b has an anti-proliferative ability in some cases. The cell cycle-regulated genes such as cyclin-dependent kinase (CDKs) and cyclin have been recognized as miR-20b target genes. In ovarian cancer, overexpression of miR-20b arrests the cell cycle in the G1 phase by reducing cyclin D1 levels [66]. The transfection of EJ cells, an invasive bladder carcinoma, with miR-20b triggers G1 phase arrest by directly targeting CDK6, CDK2, and cyclin D [76]. Furthermore, p21, a well-known inhibitor of CDKs, was indirectly increased in miR-20b-transfected cells and thus reinforced cell cycle arrest.

### Metastasis

Cancerous cells acquire several properties and transform into metastatic cells, which can migrate from the original location to distant sites and form secondary tumors [93]. This advanced cancer stage is responsible for patients' illness severity and death. Compelling evidence has indicated that various steps, such as epithelial-mesenchymal transition (EMT), migration, invasion, and angiogenesis, are involved in metastasis.

#### EMT

Reversible and rapid modulation of phenotype transition from epithelium to mesenchyme is termed EMT. During this peculiar process, primary tumor cells lose their surface adhesion molecules and obtain migration capabilities [94]. Many experiments have recognized the different signaling pathways, including transforming growth factor beta (TGF-β) participating in EMT [95]. In prostate cancer, the TGF-β signaling pathway significantly promotes EMT, while miR-20b mimic represses EMT by targeting the receptor of TGF- β [68]. The microRNA mimic approach has focused on using synthetic miRNA-like fragments for gene silencing [96]. Furthermore, incorporating long-noncoding RNA with miRNAs to regulate biological processes has become a field of interest for investigators. LncRNA operates as a miRNA sponge that binds to the desired miRNA with high affinity and inhibits their downstream functions [97]. H19, a long non-coding RNA, interacts with miR-20b and suppresses its biological function [84]. Mechanistically, miR-20b downregulates HIF-1 alpha, while downregulation of miR-20b activates hypoxia signaling pathways and stimulates EMT in endometrial cancer [84].

#### Migration and invasion

Cell migration typically occurs in embryonic development, nervous system formation, wound healing, vascular sprouting, and immune-cell trafficking [61]. The migration enables cells to change their location in either tissue or among various organs. Uncontrolled cell movement is related to pathological circumstances, including the invasion behavior of tumor cells. In invasion, malignant cells can penetrate tissue and vascular barriers into the bloodstream. Characterizing cancer cells' migration and invasive potential and interaction with various underlying regulation mechanisms, such as miRNA, is relevant for developing therapeutic strategies against cancer.

The results of the microarray assay emphasize miR-20b key role in the miRNA-mRNA network in cervical cancer migration [69]. Cervical cancer is a prevalent malignancy in women after breast and intestine cancer [98]. Papillomavirus (HPV) infection may trigger tumorigenesis and cervical cancer progression. This virus increases miR-20b levels and leads to migration and invasion via targeting tissue inhibitor of metalloproteinases 2 (TIMP2, an inhibitor of MMP2 [69]. Metalloproteases (MMPs) such as MMP2 are complicated in the degradation of the extracellular matrix, thus enabling cell migration [99]. There is another relevance between changed miR-20b levels and MMPs in bladder cancer occurrence and progression. It has been indicated that miR-20b expression is reduced in bladder tumor cells, and elevated miRNA levels can act as a tumor suppressor [76]. MMP2 is described as the potential target of miR-20b [76]. Downregulation of miR-20b predominantly yields sustained migration and invasion of bladder tumor cells.

The miR-20b also participates in tumorigenesis and esophageal cancer progression. Indeed, miR-20b activity brings about esophageal cancer migration and invasion through the downregulation of PTEN levels [71]. The regular role of PTEN is suppressing migration through its phosphatase activity [100]. PTEN inactivates the phosphoinositide 3-kinase (PI3K) signaling pathway, which is vital to provide a front-rear gradient of molecular and chemotaxis levels for cell migration. Consequently, targeting PTEN augments PI3K signaling pathway and cancer cell migration.

#### Angiogenesis

Angiogenesis is the formation of blood vessels to supply cancer cells within the tumor. As a result of its unique capacity to inspire new blood vessel formation, angiogenesis plays an essential role in tumor formation and metastasis [101]. The miRNAs regulate several angiogenesis-related signaling pathways and master genes [102]. Vascular endothelial growth factor (VEGF) is secreted by various cells, such as cancer cells, to stimulate angiogenesis. Targeting VEGF either directly or indirectly through miR-20b leads to down-regulation of angiogenesis. However, the interaction between long non-coding RNAs and miR-20b leads to the induction of VEGF expression in some cases. Enhanced levels of lncCAMTA1, a long non-coding RNA, are found mainly in the MDA-MB-231 breast cancer cell line. Further analysis indicated that lncCAMTA1 binds to miR-20b, thus up-regulating VEGF level as the miR-20b target gene [62]. In addition, VEGF downstream signaling pathways such as MAPK, ERK, Janus kinase (JAK), and Signal transducer and activator of transcription (STAT) are up-regulated in MDA-MB-231 cells, and angiogenesis occurs. Interestingly, miR-20b can target the upstream regulator of the VEGF gene and trigger angiogenesis in hepatocellular carcinoma [81]. STAT3, as the miR-20b target, negatively regulates VEGF in mRNA and protein levels. Overexpression of miR-20b in hepatocellular carcinoma cells inhibits STAT3 function, leading to increased VEGF levels [81].

#### Cancer stem cells

Recent developments in miRNA-mRNA interaction networks have led to an interest in miR-20b in cancer stem cells (CSCs), believed to play a critical role in tumor progression and drug resistance [103]. The miR-20b may be a double-edged sword in cancer stem cell regulation.

The stemness of cancer stem cells that causes the malignancy of colorectal tumor have been explored [73]. The expression of Oct4, a stem cell marker, and MALAT1, a long non-coding RNA, are negatively related by miR-20b levels. Overexpression of miR-20b attenuates the proportion of cancer stem cells via the direct targeting of Oct4 and MALAT1, critical positive regulators of cancer stem cell stemness [73, 81, 102, 103], and finally eases tumor regression [104]. However, miR-20b could promote breast cancer stem cell proliferation and cell cycle [64]. It indirectly elevates Cyclin D1 and E2F1 mRNA levels and promotes cell proliferation in breast cancer stem cells [64]. Cyclin D1 is a crucial regulator of cell cycle progression from G1 to the S phase [104]. Overexpression of cyclin D1 and E2F1 mediates the high rate of cell cycle and division [105]. Further, an examination has revealed that miR-20b may inhibit cyclin D1 and E2F1 inhibitors, ultimately increasing cyclin D1 and E2F1 expression.

### Autophagy

Autophagy is a conserved self-degradative cellular procedure to remove damaged organelles, intracellular pathogens, and misfolded proteins [106]. This fundamental process balances cell energy and response to nutrient stress [106]. The relevance of autophagy in early- and advanced cancer remains disputable. While investigations have illustrated that autophagy inhibits tumorigenesis by suppressing tumor cell growth and inducing apoptosis, it may also cause tumor cell migration and invasion [107, 108]. Depending on a wide range of regulators, including miRNAs, the role of autophagy can be different in cancer. It has been shown that miR-20b can downregulate autophagy via inhibiting RB1CC1/FIP200, autophagosome formation-related proteins [63]. Then breast cancer cells' survival decreased. Further research should be carried out to establish closer links between miR-20b and autophagy in cancer.

### Apoptosis

Apoptosis is called programmed cell death (PCD), mainly containing intrinsic and extrinsic pathways. Each pathway requires a specific initiator caspase protein which activates the downstream molecular cascade and induces apoptosis [109]. Improper apoptosis, either activation or suppression, is a key factor in human diseases like cancer [110]. A strong relationship between miR-20b and apoptosis has been reported in ovarian cancer. Increased miR-20b levels remarkably down-regulate B-cell lymphoma 2 (Bcl2) as an anti-apoptotic protein [65]. As a result, Bax protein is oligomerized on the mitochondria surface and causes induces apoptosis by releasing cytochrome c [65]. Furthermore, Increased miR-20b levels induce apoptosis in renal cell carcinoma. MAPK signaling pathway components are considered miR-20 putative targets based on the bioinformatic assay [82]. Depending on cell type, the MAPK cascade has a dual role in inducing or inhibiting apoptosis [111]. Although the MAPK signaling pathway is up-regulated in renal cancer cells and inhibits apoptosis [112], over-expression of miR-20b could inhibit this axis and induce apoptosis.

The miR-20b can inhibit apoptosis. The expression of miR-20b and its underlying mechanism of action has been investigated in cancerous thyroid cells. LncRNA- miRNA crosstalk can regulate apoptosis by affecting associated signaling pathways. double homeobox A pseudogene 8 (DUXAP8), a lncRNA, suppresses apoptosis in papillary thyroid tumor cells. Indeed, DUXAP8 binds to miR-20b and inhibits its function [78]. SOS-1 of the ERK signaling pathway, which causes cell survival, is known as the miR-20b target. Mechanistically, miR-20b inhibition by DUXAP8 improves cell proliferation while impeding apoptosis [78].

### Drug resistance

Drug resistance is the main challenge in cancer treatment, primarily preventing chemotherapy's efficacy in cancer cells and promoting cancer cell survival. MiR-20b has an essential role in chemotherapy resistance. It has been demonstrated that the activation of Syndecan-2, a transmembrane receptor, is positively correlated with chemotherapy resistance [75]. Mechanistically, miR-20b diminishes the resistance of cancer cells to chemotherapy by targeting Syndecan-2 [75]. Its capability to regulate the sensitivity of cancer cells to chemotherapy drugs through hypoxia signaling pathways has profoundly been displayed.

Interestingly, the downregulation of miR-20b in gastric cancer induces drug resistance [83]. Hypoxia-inducible factor 1 subunit alpha (HIF1A) negatively correlates with miR-20b levels in gastric cancer. As a result, increased levels of HIF1A activate downstream genes like multidrug resistance (MDR) and facilitate drug resistance [83].

An investigation of hepatocellular carcinoma cells has indicated that drug resistance negatively associates with reduced levels of miR-20b [80]. Overexpression of miR-20b leads to the sensitivity of hepatocellular cancer cells to chemotherapy drugs. From the molecular aspect, the cell division cycle 37-like 1 (CDC37L1) gene enhances drug resistance of hepatocellular cancer cells [80]. Targeting CDC37L1 by miR-20b reverses its effects. CDC37L1 is a cochaperone protein that promotes cell survival [113]. Besides, miR-20b can induce apoptosis of drug-resistance colon cancer cells by suppressing ADAM9 (A disintegrin and a metalloprotease 9), an activator of MMPs [72]. Silencing of ADAM9 expression encourages apoptosis in various tumor cells, such as ovarian and prostate cancer [114, 115].

## The miR-20b as a biomarker

Biomarkers are an increasingly important area in biomedical science [116]. These measurable indicators are biological molecules that can be found in body fluids, including blood, plasma, urea, and semen, as well as related tissue biopsy. Biomarkers provide essential information on either normal or abnormal cellular function [116]. Due to identifying miRNAs in biological fluids, miRNAs could be applied as potential biomarkers for a wide range of abnormalities such as cancer [117]. Using miRNAs as a biomarker can evolve the prognosis of malignant cancers and make it possible to evaluate tumors and the treatment protocols' efficacy.

A study on renal cell carcinoma determined that miR-20b was downregulated during tumorigenesis. Consequently, tumor cell proliferation and cell cycle were increased [82]. Therefore, miR-20b may be a biomarker for prognosis prediction and early detection of renal cell carcinoma. Besides, an elevated level of miR-20b found in breast cancer patients' serum and plasma has a positive correlation with tumor malignancy [118]. Consequently, it has the potential to be considered a biomarker with significant sensitivity and specificity. Evaluation of miR-20b level in plasma samples of endometrial cancer patients showed that this miRNA has prognostic value in advanced tumors compared to the early stage [119]. Up-regulated miR-20b in HPV- associated oropharyngeal carcinoma specimens is negatively correlated with patient survival [120], and elevated miR-20b level is associated with the shorter survival rate of breast cancer patients [121].

A recent study in breast cancer patients exhibited down-regulated levels of miR-20b in procured exosomes from tumor biopsy samples with recurrence [122]. Interestingly, exosome-packaged miRNAs are secreted into the cell microenvironment to regulate target cells and underlying signaling pathways [123].

## Conclusion

The abbrent expression of miR-20b affects tumor-related signaling pathways, underlying genes, and biological functions. Despite acting as an oncogene and tumor progression factor, miR-20b has tumor suppressor function in cancers. This dual role of miR-20 may depend on target genes in specific cellular conditions. Furthermore, integrating miR-20b function with long non-coding RNAs such as DUXAP8 in regulating fundamental cellular processes leads to inconsistent effects. Different vital tumor-related genes, such as PTEN, VEGF, MMP2, Oct4, and ADAM9, were defined as miR-20b targets. Many signaling pathways, including ERK, STAT, and TGF-beta, were regulated through miR-20b in tumor cells. Multiple comprehensive analyses revealed that the miR-20b could consider as a biomarker to evaluate tumor progression and the effectiveness of therapeutic approaches. This review provides a better understanding of miR-20b function during tumorigenesis and cancer progression. More information on miR-20b would help to establish a greater degree of accuracy on this matter. Therefore, further investigations are required to investigate the interaction between miR-20b and cancer.

## Data Availability

Not applicable.
